# Comparison of face-to-face versus email guided self-help for binge eating: study protocol for a randomised controlled trial

**DOI:** 10.1186/1745-6215-15-181

**Published:** 2014-05-22

**Authors:** Paul E Jenkins, Amy Luck, Alison Burrows, Nicky Boughton

**Affiliations:** 1 Cotswold House Eating Disorders Service, Oxford Health NHS Foundation Trust, Warneford Hospital, Oxford, UK; 2 University of Birmingham, Edgbaston, Birmingham, UK

**Keywords:** Guided self-help, Eating disorders, Email, Clinical trial

## Abstract

**Background:**

Guided self-help is a recommended first-step treatment for bulimia nervosa, binge eating disorder and atypical variants of these disorders. Further research is needed to compare guided self-help that is delivered face-to-face versus via email.

**Methods/Design:**

This clinical trial uses a randomised, controlled design to investigate the effectiveness of providing guided self-help either face-to-face or via e-mail, also using a delayed treatment control condition. At least 17 individuals are required per group, giving a minimum *N* of 51.

**Discussion:**

Symptom outcomes will be assessed and estimates of cost-effectiveness made. Results are proposed to be disseminated locally and internationally (through submission to conferences and peer-reviewed journals), and will hopefully inform local service provision. The trial has been approved by an ethics review board and was registered with ClinicalTrials.gov NCT01832792 on 9 April 2013.

## Background

Psychological therapy is the recommended treatment for eating disorders characterised by recurrent binge eating, with evidence-based self-help recommended as a first step of treatment [1]. However, few studies have looked at how best to deliver this form of treatment, although outcomes and treatment adherence appear better when guidance is available from a healthcare professional (for example, [2-4]), known as guided self-help or GSH. Furthermore, GSH has demonstrated particular cost-effectiveness, even when highly trained clinicians are used to administer the treatment [5], and a number of other RCTs have supported the utility of cognitive behaviour therapy (CBT)-based self-help in the treatment of recurrent binge eating (see [6] for a review). Typically this has been delivered face-to-face, but email-assisted therapy has been suggested as a helpful way of obtaining professional treatment without the need for face-to-face contact that is popular with patients and therapists [7]. It is potentially less costly for health service providers, and may be more acceptable to certain subgroups of patients, for example, those who might not wish to attend face-to-face appointments [8]. As eating disorders services often cover large populations, many individuals are required to make a long commute in order to attend appointments (for example, [9]) and so treatments delivered over the Internet can circumvent many of the difficulties associated with this. Similarly, treatments that require less face-to-face contact can be perceived as more empowering (for example, [10]) and are more cost-effective [11].

Guided self-help also has potential beyond specialist eating disorders (ED) services. Högdahl *et al*. [12] argue that bibliotherapy with email support has a similar content but is less costly than CBT and other Internet-based approaches. They argue that if effectiveness of such an approach can be established, it can be used in both specialist and non-specialist settings “due to minimal therapist training and low cost” (p. 38; see also [13]). In the UK, for example, Improving Access to Psychological Therapies (IAPT) is a programme within the National Health Service (NHS) that supports delivery of recommended guidelines for anxiety and depression and has more recently expanded to treat other mental illnesses and long-term physical health conditions [14]. Guided self-help significantly contributes to the interventions delivered by IAPT services [14], although its application to eating disorders (and adults in particular) is not uniform. Given that EDs are associated with significant disability and cost-of-illness (for example, [15]) - both areas targeted by the IAPT programme- the IAPT service may be well-placed to support the delivery of GSH to sufferers of some of these disorders (for example, see [16]). Although limited data exist on the effectiveness of GSH in primary care, with significant early termination of treatment (for example, [17]), rates of bulimia nervosa (BN) and binge eating disorder (BED) appear to be amongst the highest seen in affected groups. Only a minority of sufferers seek professional help and therefore being able to offer a variety of methods of delivering help may go further to reducing the devastating impact of these illnesses.

A previous randomised controlled trial (RCT) [18] compared different forms of guidance and found that, although face-to-face guidance was superior to both telephone-based guidance and self-help with minimal guidance, telephone contact demonstrated promising results and may be useful when face-to-face contact proves difficult to arrange. A more recent RCT [19] compared Internet-based GSH to a waiting list control for individuals with BN or BED. A self-help book [20] was provided, and associated guidance took the form of email contact with a graduate psychology student. The treatment condition resulted in significant symptom improvement compared to a waiting list control. This was maintained at six-month follow-up and appeared comparable to results of face-to-face treatment in other studies, although no direct test of this was carried out. A recent study by Wagner *et al*. [21] compared GSH and an Internet-based CBT program with therapist support in a sample of 155 females with BN or atypical BN. The two treatments were found to be comparableand effective in reducing ED symptoms at end-of-treatment and follow-up up to 18 months.

In addition to documented efficacy in favour of self-help, there is some emerging evidence that GSH for normal-weight binge eating disorders (that is, BN and BED) can be provided through methods other than face-to-face contact with a therapist (the term ‘therapist’ is preferred here; see below). There have, however, been few controlled studies directly assessing and comparing the effectiveness of different treatment modalities, and a recent review concluded that there exists a “lack of direct comparisons of Internet-based treatments to face-to-face treatments” for eating disorders [22, p. 549]. Only a small number of studies have compared different forms of guidance in GSH, and only one study has looked at email support compared to a control condition [19]. One RCT comparing Internet-delivered CBT versus face-to-face group CBT [22] is currently ongoing. Further research is needed to identify the efficacy and feasibility of different forms of guidance in order to develop cost-effective and beneficial treatments that can be adapted to patient need.

### Objectives and hypotheses of the RCT

The overall aim of the trial is to evaluate whether therapist support within GSH can be as effective when delivered via email as when delivered face-to-face. In this paper, we detail the design and methods of the current trial, and clarify the four main aims of the study. Specifically, these are to: (1) compare the effectiveness of face-to-face versus email GSH during treatment and at follow-up; (2) determine relative attrition and drop-out across the two treatments; (3) look at predictors of outcome in GSH (see below); and (4) determine the relative cost-effectiveness of these two treatments. The study also employs a waiting list control condition. The current paper describes the protocol on which the clinical trial is based, in line with suggested guidance (for example, [23]).

In terms of the primary outcome measure, it is expected that both treatment conditions will be superior to the waiting list control condition (for example, [4,18]). Although there is a limited amount of evidence in eating disorders, it is hypothesised that both treatments (face-to-face and email GSH) will be comparable in terms of effectiveness (for example, [24]) in reducing binge eating frequency (see also [21]). It is expected that similar results will be found with secondary outcome measures, drop-out and cost-effectiveness, although these variables have not yet been studied in detail in this way.

## Methods and design

### Study setting and trial design

The trial is a three-arm, individually randomised, controlled trial with two active treatments and a waiting list control. The study is being conducted within Oxford Health NHS Foundation Trust in the United Kingdom. The Eating Disorders Service covers a population of around 1.6 million adults and has three sites, each of which is recruiting participants as part of the trial. Participants are randomly assigned (using a random number generator) to one of three conditions: face-to-face GSH, email GSH or a waiting list control condition. Each condition lasts 12 weeks, and follow-up evaluations are conducted six months post-treatment. Ethical approval for this Trial was obtained from NRES Committee South Central-Oxford B, National Research Ethics Service, Reference: 13/SC/0217. The trial is registered with ClinicalTrials.gov, no. NCT01832792. Participants receive verbal and written information before consenting to the study, are asked to indicate written consent (after at least 24 hours to consider this), and are instructed of their right to withdraw at any time. The research was conducted in compliance with the Declaration of Helsinki and Good Clinical Practice (GCP) and will be reported in line with Consolidated Standards of Reporting Trials (CONSORT) guidance.

### Eligibility criteria

To be eligible for participation, individuals registered with a General Practitioner covered by Oxford Health NHS Foundation Trust must: (1) demonstrate regular binge eating (defined as either subjective or objective bulimic episodes) in the context of an eating disorder; and (2) be aged 17.5 or older. Participants are excluded from the trial if they reported any of the following: (1) body mass index (BMI) < 18.5; (2) recent rapid weight loss (defined at >1 kg/week); (3) any major medical condition that might interfere significantly with treatment; (4) current excessive drug or alcohol use; and (5) active and untreated psychosis or severe depression. Other contraindications to GSH (such as developmental disability that would impair the ability to participate in the treatment, or severe and complicated EDs) also represent exclusion criteria.

### Participant information sheets

Participant Information Sheets were approved by the Research Ethics Committee (REC), and provide an overview of the study for potential participants. Researchers are also on hand to explain the information contained in the information sheet and to ensure fully informed consent, documented with signed consent forms. Participants are not deceived in any way. Readability of information sheets was also considered and assessed using the SMOG Grading index [25]. The SMOG was calculated using an online calculator developed by its author (http://www.harrymclaughlin.com/SMOG.htm) and can generate equivalent grade levels for comprehension, which can also be categorised in line with the United States Department of Health and Human Services (USDHHS) standards (for example, [26]). SMOG levels and equivalent classifications and school ages for the participant materials used in the present study are reported in Table 1.

**Table 1:** Readability estimates for participant information sheets

**Material**	**SMOG score**	**USDHHS classification**	**British school age equivalent**
**Participant information sheet**	10.27	Difficult	15 to 17 years
**Consent form**	10.19	Difficult	15 to 17 years
**Email security information sheet**	9.24	Average	12 to 15 years

### Interventions

All eligible patients are offered participation in the trial following a team discussion within the Eating Disorders Service (EDS) and are then randomly assigned to one of the three conditions. This involves meeting with one member of the research team who will go through the trial protocol and information sheets. This is not the patient’s individual clinician, but the appointment can potentially occur directly before or after a planned appointment if this is more convenient for the participant. This helps ensure completion of questionnaires and enables the participant to ask any questions directly relating to the trial. If individuals decide not to participate in the trial, they are offered treatment as usual.

Both GSH interventions are based on a self-help treatment [20,27] that has documented efficacy, and a copy of the book is provided to patients. The treatment is “program-led” [28]; that is, the aim of the therapist is to support the patient in adhering to the principles of the book, and thus does not require extensive clinical training. The treatment is designed so that non-specialist therapists can readily administer this intervention [29].

### Face-to-face GSH

This treatment follows the self-help manual, *Overcoming Binge Eating*[20,27], and provides 10 sessions of face-to-face therapist care, with each session lasting 20 to 25 minutes. The first session may last longer than this, due to a need to review since the patient was last seen at the EDS and to allow the patient more time to socialise to treatment (see [28]). Guidance for the provision of GSH has been provided in Fairburn [28], and 10 sessions are provided over a period of 12 weeks. There is no specific guidance about the frequency of sessions, although the initial sessions are recommended to be at least weekly with the remaining sessions being adapted to suit the patient’s progress [28]. A typical format might be to have the first eight sessions weekly, with the remaining two occurring over the following month (that is, fortnightly).

The second edition of the book in particular [27] might be considered as ‘multicomponent GSH’ as it has been based on Cognitive Behaviour Therapy-Enhanced (CBT-E) [30] and designed to address many core elements of an eating disorder, including topics such as self-monitoring, psychoeducation and regular weighing. The support provided in this RCT is, adopting the taxonomy suggested by Glasgow and Rosen [31], likely to fall somewhere between ‘therapist-administered’ and ‘minimal contact’. A more recent paper [32] has elaborated further on this taxonomy, suggesting that the form of GSH used here is best described as ‘minimal contact therapy’ as opposed to ‘therapist-administered’, the latter being where self-help material “augments the impact of a standard therapy” (p. 90,). The recent review of Farrand and Woodford [33] found a lack of studies adopting this taxonomy, although the definition suggested by Glasgow and Rosen [31] (and the more updated modifications from Newman *et al*. [32]) might be difficult to determine *a priori*. Many evaluations of self-help treatment have considered both of these categories together under the wider umbrella of ‘GSH’ and, while the lack of definition regarding the degree of involvement from professionals is a shortcoming of the existing literature [33], it is hoped that a retrospective assessment of this can be made in the current study. It would be interesting to look at the content of GSH sessions retrospectively to consider the degree of support (that is, ‘therapist-administered’ or ‘minimal contact’) as a possible moderator of outcome. In terms of treatment in the current study, GSH was devised by the developer to involve a therapist, but to a lesser degree than in conventional CBT (see [28]). The treatment was designed to be used by non-specialist therapists who help the patient follow the GSH programme [29] by providing support, encouragement and monitoring of progress. Therapists also discuss goals and elements of the programme identified by patients as those that they wish to raise (for example, parts they donot understand, disagree with or parts that are not especially relevant), as well as potential solutions.

Of note, the term ‘therapist’ is used here to refer to those individuals on the ‘professional side’ of the patient-therapist dyad. However, some authors (for example, [34,35]) have argued that use of this term in self-help could set up certain expectations about the treatment that might hamper progress; thus, terms such as ‘guide’ and ‘facilitator’ have alternatively been used. As in other treatments and manuals the precise term used could be modified to suit the individual setting (for example, see [36]).

### Email supported GSH

This treatment involves patient-therapist contact via e-mail. Patients are advised to follow the treatment closely (which is divided into ‘steps’) and instructed to contact the therapist at least once a week, and can receive feedback up to twice a week for a period of 12 weeks [19]. Email is the preferred medium for all communication, with face-to-face and telephone appointments occurring at a minimum. The one exception to this is that the first appointment is always conducted in person so that the patient can meet their therapist, receive a copy of the manual, be weighed at the clinic and so on. As sparse guidance exists on how best to implement email supported GSH, the treatment has been conceived firstly to parallel the face-to-face condition (see above); for example, contact is less frequent towards the end of the sessions. We have, secondly, followed the methodology of Ljotsson *et al*. [19] as this was the first example of providing GSH with the current manual with email support. Participants could (although this is not an exclusive list): get feedback on homework and progress; ask questions; and receive guidance regarding the self-help program. A more recent study [12] has described a similar intervention, although less detail is reported on the exact nature of support. The focus of supervision and the practice of treatment were to follow the guidelines of the therapist’s manual [28]. In the current study, therapists are advised to follow similar guidelines to the face-to-face condition. For example, therapists will still aim to discuss problems, identify solutions and monitor progress; the treatment is proposed to be delivered in a similar manner, but via the medium of email. The focus in both treatment conditions is on the eating disorder, and therapists may only address other issues if they are of “immediate relevance to the binge eating problem” ( [28], p. 9). Email can be sent securely by both patient and therapist although patients will have to agree to download a software package from Cisco and create a password (this is optional, although it enhances data security). Identification of any significant increased risk (for example, medical, suicidal) would prompt a telephone call or face-to-face meeting from the therapist.

### Delayed treatment condition

The delayed treatment condition lasts 12 weeks, and data obtained from this period serve as waiting list control conditions. As at other stages of treatment, if, at review, participants wish to opt out of the study they will receive treatment as usual. There is no six-month follow-up for this condition.

### Primary and secondary outcomes

The primary outcome will be frequency of objective bulimic episodes, as assessed by the Eating Disorder Examination Questionnaire (EDE-Q) (see below). Comparisons will be made between pre-treatment, post-treatment and follow-up in the intervention groups. Secondary outcome measures include overall eating psychopathology, other ED behaviours, such as self-induced vomiting, psychological distress, self-esteem, functional impairment and healthcare usage, assessed by self-report (see below). Some of these variables (for example, severity of ED psychopathology) will be considered as predictors of outcome, as well as considered aspossible mediating or moderating variables, such as therapeutic alliance or type of therapeutic support.

### Assessments and timeline

All patients are assessed by the EDS following initial referral to the service. This is a full clinical assessment and will identify current symptoms as well as aspects related to comorbidity, risk and so on. For example, if patients indicate on self-report questionnaires that they are a risk to themselves or others, the clinician will discuss this further. If they are then eligible for, and consent to, the trial they meet with one of the research team to give written consent. The assessment procedure is outlined in Table 2.

**Table 2:** Schedule for assessment

**Measure**	**Baseline**	**Session 3**	**End-of-treatment (12 weeks)**	**Six-month follow-up**
***Self-report***				
**Demographics form**	X			
**Contact information**	X			
**EDE-Q**	X		X	X
**CORE-OM**	X		X	X
**RSES**	X		X	X
**CIA**	X		X	X
**Helping alliance questionnaire**		X	X	
**Healthcare usage questionnaire**	X		X	X
***Completed by therapist***				
**Weight/BMI**	X		X	X
**Therapist time questionnaire**			X	X
**Helping alliance questionnaire**		X	X	

BMI, body mass index; CIA, Clinical Impairment Assessment; CORE-OM, Clinical Outcomes in Routine Evaluation - Outcome Measure; EDE-Q, Eating Disorder Examination Questionnaire; RSES, Rosenberg Self-Esteem Scale.

Data from the following measures will be collected:

The EDE-Q v6.0; [37] is a self-report questionnaire that assesses eating disorder symptoms and has good psychometric properties [38]. Participants rate how frequently they have experienced symptoms (both attitudinal and behavioural) of an ED over the last month on a 0 to 6 scale (ranging from “no days” to “every day”).

The Clinical Outcomes in Routine Evaluation-Outcome Measure (CORE-OM; [39]) is a self-report measure of general psychological distress. Thirty-four items assess symptoms over the preceding week and are rated on a five-point frequency scale (from “not at all” to “most or all of the time”), which can generate a global score indicating overall functioning. Its utility in eating disorders has been demonstrated [40].

The Rosenberg Self-Esteem Scale (RSES; [41]) comprises 10 items that measure global self-esteem. Responses are given on a four-point scale from 1 (“strongly agree”) to 4 (“strongly disagree”), with half of the items reverse-scored.

The Clinical Impairment Assessment (CIA; [42]) is a 16-item self-report measure assessing psychosocial impairment resulting from ED symptoms. Participants are asked to consider the preceding 28 days and to indicate to what degree symptoms of an ED have affected different areas of their life. Responses range from “Not at all” to “A lot” (scored from 0 to 3), and a total score (0 to 48) can be calculated giving an overall indication of functional impairment. The CIA has demonstrated acceptable psychometric properties across a range of samples [43].

The Helping Alliance questionnaire (HAq-II; [44]) is a 19-item measure of therapeutic alliance, showing good psychometric properties. It comprises both positive and negative items (which are reverse-scored), and includes both a patient- and therapist-version. Statements regarding the therapeutic alliance are rated from 1 (“strongly disagree”) to 6 (“strongly agree”). A mean score is calculated by adding up all items (reverse-scoring as appropriate), calculating a mean, and then multiplying this by 19 (which should be identical to the total score if all items are completed). Thus, scores range from 19 to 114 (with higher scores indicating better alliance). It has the advantage of assessing alliance independent of symptom improvement and has been used in previous ED treatment studies (for example, [45]).

In addition, a measure assessing healthcare use developed specifically for this study will be given (see Additional file 1). This will be administered with the standard pack of questionnaires (that is, at start-of-treatment, end-of-treatment and follow-up) for individuals who consent to the study. An estimate of cost effectiveness will be made by monitoring resource use directly associated with different treatment conditions (for example, therapist time spent providing care). The proportion of binge-free days (estimated through the EDE-Q) will also be used to estimate cost effectiveness (for example, [46]). Therapist time will be estimated using a simple checklist of time spent engaged in treatment-related activities. The measures listed should allow comparison with other studies [33] and also overcome some of the shortcomings in previous studies, such as limited estimates of cost-effectiveness. Self-report measures may also limit observer bias, although this may be open to subject bias. The lack of a structured clinical interview (for example, the Eating Disorder Examination) is also a shortcoming, although patients are screened on the basis of meeting criteria for an eating disorder, following an interview with an experienced clinician.

### Sample size

A power calculation (using an α(two-tailed) of 0.05, an anticipated effect size of 1.0 and a desired power of 0.8) suggested that a minimum number of 17 individuals per group [47], thus a minimum N of 51 is required. This was based on the findings of Ljotsson *et al*. [19], who reported a reduction in Global EDE-Q scores during self-help treatment of 1.1 (*SD* = 0.9). Notable variability in effect sizes for GSH treatment has been reported, with a recent systematic review of self-help interventions finding an overall effect size of 0.54 (95% CIs 0.36 to 0.72) across eight studies [33] and another [48] citing effect sizes for the frequency of binge eating ranging from .03 to 2.68. Effect sizes in such studies have been shown to be affected by factors such as level of pathology, sample (for example, community vs. clinical), and length of follow-up [33]. Therefore, we are aiming for a minimum N of 51, but ideally closer to 44 per group [47] to account for this variability and to minimise the risk of the study being under-powered. Thus, a minimum overall recruitment sample of 102 is sought, estimating dropout at 50%, slightly less than the 55% reported by Loeb *et al*. [3] and higher than the approximately 23 % reported by Ljotsson *et al*. [19]. Attrition rate has been considered as an important factor in evaluating such trials [48]. Therefore, in the current study, an attempt is made to more clearly define individuals who do not complete treatment, for example, noting at what point participants ceased treatment (for example, [49]).

### Allocation

Participants who have given written consent to participate will be randomly assigned to a waiting list, face-to-face GSH, or email GSH condition, with a 1:1:1 allocation as per a computer-generated random number sequence. Numbers are stratified (for example, 0 to 0.333) and correspond to allocation to a treatment arm. If participants in the waiting list condition wish to remain in the trial, they will then be randomised to one of the two treatment conditions using the same method, but with a 1:1 allocation. In order to further enhance allocation concealment, the allocation for the first cohort of cases (the number is not disclosed here) has been determined *a priori*. If more individuals than this are recruited, the procedure will be repeated. Reviews have highlighted unclear randomisation procedures in many RCTs [33], and this method should limit predictability of allocation due to use of “a random and unpredictable assignment sequence” ( [50]; see also [51]). Although this was conducted by the Chief Investigator (CI) (rather than an external researcher), sequence generation was completed prior to the first participant being recruited and participants are allocated only after full informed consent is received. In line with GCP guidance, full consent is obtained at least 24 hours after meeting with a member of the research team, and thus treatment allocation is not known at the time of recruitment, keeping allocation concealed until treatment is assigned [50].

### Data collection

Data from the EDE-Q provide the primary outcome variable - binge eating frequency. Secondary variables include compensatory behaviours (for example, self-induced vomiting), and ED psychopathology, as well as constructs assessed by the other measures (self-esteem, psychological distress, functional impairment). Weight and BMI are not outcome variables, but may be included in analyses, for example, to give demographic information or as covariates. Other routine demographic information (for example, ethnicity) will also be recorded. Participants are asked to complete self-report measures (mailed or otherwise given by hand) at start-of-treatment, end-of-treatment and six-month follow-up (with the exception of the HAq-II; see Table 2). Data will be stored on a secure computer server and analysed with computer statistics software (for example, SPSS **IBM Corporation, Armonk, NY, USA**). Oxford is the lead site for data coordination. In order to promote data quality, data will be examined for impossible values and a sample of electronic data (around 10%) will be double-checked by another researcher with raw data. Blinding is not used in this study, and so an emphasis on appropriate outcome measurement and allocation concealment is crucial [50,51].

### Statistical methods

Regarding the primary aim - comparing active treatment and control condition - results will be analysed using analysis of variance and *t*-tests. Effect sizes will also be assessed, and expressed appropriately (for example, Cohen’s *d*). Logistic regression analyses will look at predictors of outcome, and potential mediators and moderators might also be considered. However, a number of existing studies addressing this issue have been underpowered [33] so this remains an important consideration. Differences in outcome variables will be analysed (for example, through χ^2^, regression) to examine attrition/drop-out. In terms of cost-effectiveness, analyses will be conducted based on data obtained through the health utilisation questionnaire, therapist time questionnaire and measures of functional impairment (CIA) and ED symptoms (EDE-Q).

#### Therapists, training and supervision

All therapists will treat patients in both conditions, and comprise individuals from a number of professions, such as psychology and nursing. All have experience working with individuals with eating disorders but, in line with the suggestions regarding GSH (for example, [28]), they are not particularly experienced in providing psychological therapy. Therapists in the current study comprise both those with a professional healthcare qualification (for example, nursing) and paraprofessionals (for example, support workers, health care assistants), all of whom have at least an undergraduate degree plus one year of experience working in mental health care. The term ‘therapist’ is used here for simplicity to encompass all of those providing treatment within the trial. Fuller details of the therapists involved will be reported following study completion, in part due to the duration of the study and probability that the workforce may change over the course of the study; therefore, it is impossible to say at this point precisely who will comprise the personnel.

The training and background of therapists has also been identified as an issue in therapist-administered treatments [52-54]. In the current trial, all therapists are specifically trained in the use of GSH, which involves extensive reading of the book, consultation of the therapist manual and supervision with the CI, a clinical psychologist experienced in the provision and supervision of GSH. As in the original effectiveness study [29], supervision is closer for a therapist’s first few cases. Therapists are recruited from the local NHS (Mental Health) Trust and no prior experience in working with EDs is required. Most are likely to be recruited from the Eating Disorders Service providing the treatment, although therapists from other services (for example, Child and Adolescent Mental Health Services(CAMHS)) have expressed interest. Although research has found mixed results (including some finding no differences in clinical outcomes) when considering different ‘types’ of therapists, therapist factors have been found to be important, even in Internet-delivered interventions (for example, [55]), and both research and clinical practice may benefit from a focus on “what the therapist actually does” [56], p. 282. To this end, clinical notes (including email transcripts) are regularly reviewed by the supervisor, in part also to maintain treatment fidelity.

An ‘understudy’ system is in place (for example, [30]) in case of therapist absence, although this is recommended to be avoided wherever possible. All therapists are instructed to familiarise themselves completely with the treatment manual and to read specific preparatory material (such as the therapist manual). Therapists meet with the CI prior to the start of the trial in order to discuss any major concerns or to raise any questions. Their adherence to the protocol will be monitored through supervision and meetings with the Chief Investigator. Therapists keep electronic notes of each session, in addition to a record of direct time spent on patient care (for cost-effectiveness evaluation). Patients are allocated to therapists according to site and therapist availability.

Dr. Jenkins serves as the overall supervisor for the study as well as the on-site supervisor for one of the locations (Oxford). Other locations are supervised on-site by other senior psychologists with extensive experience in eating disorders. In order to pilot a novel medium of supervision, therapists receive cross-site supervision from Dr. Jenkins via an online, secure, discussion forum. Questions and responses can be posted such that all therapists involved in the study have access to this whenever they have a secure Internet connection. This also allows detailed analysis of the content of supervision for evaluation purposes.

#### Data monitoring

##### Research governance

The local REC will review the project at least annually, including safety and progress reports from the principal investigator. Any significant modifications to the protocol will be submitted for approval to the REC prior to implementation and in accordance with policies. Case report forms (CRFs) are also kept, in line with GCP principles.

##### Protocol adherence and untoward events

Non-negligent indemnity is provided by Oxford Health NHS Foundation Trust, which is responsible for monitoring the safety of any data obtained and also acts as the study sponsor. Data are kept on a secure server, which requires password access. The Trust monitors the study and has authority to review the protocol and suspend the trial, and also ensures that the study is being conducted according to the protocol. Any adverse events identified in the study are immediately reported to the Trust. A pilot study will be conducted to evaluate both the methodology and to give a more accurate estimate of sample size requirement (see [57]). A new edition of the self-help book [27] has recently been released and an amendment to the protocol submitted to the local REC has permitted use of the second edition with all but the first 15 individuals, who will form a pilot study.

##### Trial closure

The end of the trial will be the date of the last follow-up of the last participant.

##### Confidentiality

All study-related information will be stored securely, with participant information kept in locked file cabinets in areas with restricted access. Unique participant identifiers will be used (rather than names) to maintain confidentiality, although a document linking these two identifiers will be kept in case of need (for example, a participant later requesting that their data be removed). Data files including the full dataset will not include names to ensure confidentiality.

## Discussion

Binge eating and related disorders are serious health concerns that are associated with a number of psychosocial, physical and occupational impairments (for example, [58]). However, despite this and the risk of developing further complications, many individuals with eating disorders do not seek treatment. The results of this trial have the potential to offer a more flexible approach to the treatment of binge eating disorders, one that is patient-centred and, possibly, more cost-effective than existing treatments. A further aim is to evaluate the possibility of providing supervision over the Internet for eating disorder interventions, an idea that has not received much empirical attention, although some studies have used online supervision forums as part of mental health treatment (for example, [59]). As is also suggested in the recent review of Aardoom and colleagues [60], future studies would benefit from investigating therapeutic alliance across face-to-face and Internet-delivered treatments. Although some limitations of the current design exist (for example, imperfect allocation concealment, lack of blinding, lack of a placebo control (see [31]), it is hoped that the results of this methodology will produce high ecological and external validity, as this (clinical) sample is recruited through a typical UK healthcare service, and may be described as a ‘pragmatic RCT’ [51].

Although Internet-delivered interventions have promise and appear to be highly acceptable ( [60]; see also [61]), there are many potential drawbacks. Some studies report negative feedback regarding email communication (for example, [7,62]), and individuals may require additional commitment or motivation than a treatment requiring face-to-face attendance. Further, although computer literacy is increasing (that is, the “digital divide” is decreasing; [63]), some individuals do not have access to the necessary materials that may be required for email therapy (for example, [64,65]). As a number of authors have suggested, it may be the case that ‘e-therapy’ is appropriate for some, but not for others. It is hoped that the results of the current trial might go some way towards supporting therapeutic equivalence for the two treatments, and thus give patients more choice when accessing treatment.

Results of this study are hoped to inform different ways of delivering the ‘first step’ of eating disorders care. It aims to determine the relative effectiveness of email-provided care compared to traditional face-to-face care and a control condition. Furthermore, it aims to look at novel methods of supervision and to evaluate the cost-effectiveness of these interventions. In this way, it is hoped that clear directions for future research and care provision might be identified, ideally those that offer patients with eating disorders the most effective treatments, in a manner that suits them, with the best economic value.

## Trial status

The trial is now recruiting (as of August 2013), and is scheduled to continue until July 2017.

## Abbreviations

BED: Binge eating disorder; BMI: Body mass index; BN: Bulimia nervosa; CBT: Cognitive behaviour therapy; CIA: Clinical Impairment Assessment; CORE-OM: Clinical Outcomes in Routine Evaluation - Outcome Measure; ED: Eating disorder; EDE-Q: Eating Disorder Examination Questionnaire; EDS: Eating Disorders Service; GCP: Good Clinical Practice; GSH: Guided self-help; HAq-II: Helping Alliance Questionnaire; NHS: National Health Service; NICE: National Institute of Health and Care Excellence; IAPT: Improving Access to Psychological Therapies; REC: Research Ethics Committee; RCT: Randomised controlled trial; RSES: Rosenberg Self-Esteem Scale.

## Competing interests

The authors declare that they have no competing interests.

## Authors’ contributions

PEJ and NB conceived of the study, and participated in its design and coordination and drafted the manuscript. AB and AL offered additional comments on the design and revised the manuscript. All authors read and approved the final manuscript.

## Supplementary Material

Additional file 1 Measure to assess healthcare use. Click here for file
