# Supplementary material for: Comparison of face-to-face versus email guided self-help for binge eating: study protocol for a randomised controlled trial
Source: Trials. 2014 May 22;15:181. doi: 10.1186/1745-6215-15-181 (PMC4035724; doi:10.1186/1745-6215-15-181)
Supplement: Additional file 1 — Measure to assess healthcare use. [file 1745-6215-15-181-S1.docx]

Measure to assess healthcare use

The following questionnaire will allow us to look at how much your eating disorder impacts your life. Specifically, it asks you to indicate what health services you have used as *a direct result of* your eating disorder. You do not have to answer, but any information you can give would help us estimate the wider impact of your illness. We would like to know what has happened to you over the last 3 months.

1. How many full days have you lost from **work** due to eating disorder symptoms or concerns in the last three months? Please give your best guess (circle N/A if not applicable)

__________________________________________________________________ N/A___

1. How many full days have you lost from **school / college / university** due to eating disorder symptoms or concerns in the last three months? Please give your best guess (circle N/A if not applicable)

__________________________________________________________________ N/A___

1. How many full days of *reduced productivity* have you experienced while at **work** due to eating disorder symptoms or concerns in the last three months? Please give your best guess (circle N/A if not applicable)

__________________________________________________________________ N/A___

1. How many full days of *reduced productivity* have you experienced while at **school / college / university** due to eating disorder symptoms or concerns in the last three months? Please give your best guess (circle N/A if not applicable)

__________________________________________________________________ N/A___

1. Have you had any investigations done, such as X-Rays, blood tests, or ECGs for your eating disorder *in the last three months*? Yes □ No □

If yes, please tell us which investigations you had done.

__________________________________________________________________ _____ ___

__________________________________________________________________ _____ ___

__________________________________________________________________ _____ ___

1. Have you seen your GP because of your eating disorder *in the last three months*?

Yes □ No □

1. Have you seen any of the following for your eating disorder *in the last three months*? (Do **not** include your current therapist, if applicable)

If yes, how many times?

- Psychiatrist Yes □ No □ ___
- Dietician Yes □ No □ ___
- Physiotherapist Yes □ No □ ___
- Occupational therapist Yes □ No □ ___
- Social worker Yes □ No □ ___
- Accident & Emergency Yes □ No □ ___
- Psychologist Yes □ No □ ___
- Any other health professional Yes □ No □ ___

1. Finally, how much do you estimate it costs you to attend appointments **here**? (Please answer each question, putting “0” if it does not apply)

- Money paid directly (e.g., bus fare) [please estimate]: £.............................
- Money paid indirectly (e.g., car mileage) [please give mileage to and from appointments, and we will estimate the cost from this]: ..................................... (miles)
- Other costs (please specify):............................................................................................................. ..................................................................................................................................................................................................................................................................................................................................................................................................................................................................................................................... ........................................................................ .............................................

Thank you
